# The grounded cognition foundation of the first cognitive model in cognitive behavior therapy: implications for practice

**DOI:** 10.3389/fpsyg.2024.1364458

**Published:** 2024-03-08

**Authors:** Alexandru I. Tiba

**Affiliations:** Department of Psychology, University of Oradea, Oradea, Bihor, Romania

**Keywords:** grounded cognition, cognitive vulnerability, modal cognition, cognitive behavior therapy, rational emotive and behavior therapy

## Introduction

Cognitive Behavior Therapies (CBTs) are the gold standard therapies for emotional disorders (David et al., 2018). How cognition is structured and restructured is central to many CBTs. Based on the mainstream cognitive science model, many influential CBT forms such as cognitive therapy and rational emotive behavior therapy (REBT) have adopted an amodal perspective on cognition. From this framework, cognition is pure information transduced from experiences or experience-independent amodal symbols (language-like symbols) that are stored in memory (Barsalou, 2008). Distortions of cognition are the result of the accumulation of more negative knowledge due to the preferential processing of negative information and previous learning. Significant developments in cognitive science (Barsalou, 2008, 2020) and systems neuroscience (Barrett, 2009; Shaffer et al., 2022) have proposed a different framework for understanding cognition. In this framework, cognition is modal and closely connected to our experiences through simulations of the brain states activated during the experiences mentioned in the cognitive content.

Some notable efforts have been made to adopt different modal frameworks for cognitive models in CBT (Tiba, 2010, 2018; Gjelsvik et al., 2018; Tiba and Manea, 2018a,b; Riskind et al., 2021), but many types of CBT, especially the cognitive-oriented CBTs, are still hesitant to do so in order to better understand cognitive vulnerability to emotional disorders. This reticence may also be found in the field of REBT, a pioneering form of CBT. This reticence in REBT is surprising since, as I argue in this paper, REBT was founded on a model of cognition based on modal principles (Barsalou, 2008).

In this paper, I argue for the first time that instead of ignoring Albert Ellis's revised cognitive model (Ellis, 1996), we should think about it as a basic understanding of cognition in CBT theory and practice. I briefly describe the modal approach by focusing on the grounded cognition perspective on cognition (Barsalou, 2008). Then, I discuss two distinctive features of the cognitive model proposed by Albert Ellis: the identity between cognition, feelings, and behavior and the biological basis of cognition (Ellis, 1957). These principles suggest that the first ideas of cognitive vulnerability to emotional disorders in CBT were based on modal principles of cognition. Finally, I discuss new ways for understanding the cognitive model of emotional disorders and new interventions for cognitive restructuring brought about by using a modal framework of cognitive vulnerability in CBT.

## The modal and amodal frameworks of cognition

The amodal model of cognition considers cognition based on an amodal, language-based information representation (Fodor, 1975). According to this model, our experiences are transduced into symbolic representations stored in memory (Barsalou, 2008, 2020). Then, these representations are re-activated and influence our reactions. For instance, when we encounter an aggressive cat, we learn that cats are aggressive, and we store this knowledge in our memory. Later, when we interact with cats, we remember they are aggressive, and we react emotionally (with fear). This model still holds today, with many considering an amodal basis of cognition for psychotherapy interventions that mainly undo dysfunctional learning.

Recent advances in cognitive science suggest that our experiences are not re-written in a language-like way. Instead, they suggest that sensory modalities, action, affect, and introspection may be the basis of cognition, especially when it comes to how cognition and emotion interact (Barsalou, 2020). According to the grounded approach, cognition uses brain modality-dependent resources (grounding) depending on the environment and body-environment transactions (situatedness) (Barsalou, 2008). In this case, when we encounter an aggressive cat, those affective, sensorial, and motor experiences are captured in memory in the same systems that were active during the experience of interaction. Later, when we interact with cats and think of cats, our cognitive system re-activates the experiences we had during our interactions with cats. When these simulations are fully reactivated, we feel fear for cats and run.

## The grounded foundation of Ellis' cognitive model

Nowadays, there are many research-based cognitive models in CBT. However, Albert Ellis's first cognitive model of CBT (Ellis, 1957). Ellis (1957) advanced the first cognitive model of emotional disturbance (the ABC model) and its treatment. This was the origin of REBT, the oldest form of cognitive behavior therapy (David, 2014) and one of the most influential forms of CBT today. According to Ellis' ABC model, it is not adversity (A; adverse events) that results in unhealthy feelings (C), but rather our irrational beliefs (B). To change unhealthy feelings, one would have to first change irrational beliefs.

The ABC model proposed by Albert Ellis was based on two distinctive principles that set it apart from later cognitive models: the identity (or interdependence principle) of cognition, emotion, and behavior and the biological principle of cognition. As I discuss, these two distinctive principles characterize Ellis' cognitive model as a grounded cognition model.

## The composite belief: the identity between cognition, affect, and behavior

It has been over 20 years since Albert Ellis revised the ABC model (Ellis, 1996, 2001) and proposed a new ABC model: the revised ABC model of REBT. In the revised ABC model, Albert Ellis has conceptualized Beliefs as a composite psychological phenomenon of thinking, feeling, and behaving. According to this view, Beliefs are at the same time thinking, emoting, and behaving. When people face adversities (A) they create unhealthy emotions (C) because they react to A with destructive Beliefs (B), thinking they must obtain what they desire or otherwise it is awful and unbearable, at the same time feeling the need to change the adversity along with tension, anxiety, and agitation, and having compulsive impulses or urges to get what they want (Ellis, 2001). This definition is a definition of beliefs (that can also be feelings and behavior) and should not be confused with the cognitive part of an emotional response (C). As stated by Ellis “...people's complex thoughts-feelings-actions lead to a disturbed complex of thoughts-feelings-actions” (Ellis, 1996, p. 106).

## Why is this an argument for considering the ABC model of REBT as a grounded cognition model?

The central idea of the mainstream amodal view of cognition (beliefs) is that input, cognition, and output are separate yet interacting components. A critical experience or input (A) interacts with cognition (B) and produces emotional and behavioral output reactions (C). Accordingly, the mainstream ABC cognitive model suggests that beliefs, feelings, and behaviors are distinct components. B is an entity distinct from feelings. This is in contrast to Ellis' model, which contends that beliefs are composed of feelings, behaviors, and cognitive components. Moreover, Ellis suggested that there is an identity relationship between these components (Ellis, 1996). This approach to cognition is similar to a grounded cognition model. The grounded cognition model suggests, in line with Ellis' proposal, that cognition linked to emotion (the B) is the same as emotion. Cognition about emotion is composed of the simulations of the referenced experiences (when I think something is awful, an awful experience including emotion is a simulation for the purpose of understanding). Thus, it is an identity relationship.

## The biological foundation of cognitive vulnerability

Ellis has consistently argued for the biological basis of cognitive vulnerability. He strongly claimed that cognitive vulnerability (irrational beliefs) stems from biological tendencies (Ellis, 1957). This is opposed to the mainstream view of cognition, which suggests that cognition is rather learned. Recently, Beck integrated biological tendencies into the generic cognitive model (Beck and Haigh, 2014), yet he does not argue for a biological basis of negative knowledge. Rather, he suggested that biological tendencies influence attentional biases toward negative information, which result in increased amounts of negative knowledge. Negative knowledge stored in memory further biases responses (Tiba and Manea, 2018a).

## Why is this an argument for considering the ABC model of REBT as a grounded cognition model?

The mainstream amodal view of cognition considers cognition stored in a semantic system. Despite the fact that the semantic system is a brain system, its biological foundation has no bearing on the information's quality. Biological tendencies cannot qualitatively influence cognition. In Marr's (1982) terms, the implementation level does not interact with the algorithmic level. The grounded cognition perspective suggests otherwise. Accordingly, knowledge depends qualitatively on the state of brain systems recruited for the simulation of the content of cognition. The neural reuse hypothesis (Anderson, 2010) suggests that experiential brain systems are reused for cognition. Thus, when cognition recruits a hyperreactive affective system for representation, cognition will show an emotional exaggeration (it is neither awful nor just bad). When cognition (thinking what you can do) recruits a hypoactive motor brain system into representation, cognition will show a deficit in the motor ingredients of cognition and a motor deficit bias (I think I cannot move).

## Discussion

Our thoughts can be conscious or unconscious. Similarly, depending on the context, thoughts can be modal or amodal. Thus, the idea that a thought can be grounded does not exclude the idea that the same thought can be represented amodally. Yet, it suggests that the associated affective simulation is responsible for the emotional impact of cognition.

This article argues that the first cognitive model of REBT, a pioneering form of CBT (Ellis, 1957), was, from inception up to recent times, based on a modal approach to cognitive representations. Yet, the implications of the modal nature of this cognitive model have not been considered in practice. There are several practical implications of adopting a grounding cognition model in cognitive-oriented CBT:

(1) Ellis' proposal of cognition as *believing-emoting-behaving* composites puts cognitive vulnerability in another modal framework of cognition: the psychological construction approach (Barrett, 2009). The same distorted psychological state (e.g., nobody loves me) may be construed and experienced as a cognition, feeling, or behavior, depending on the main ingredients, the focus of attention, and associated concepts that were activated at that moment (Barrett, 2009). Although considering cognition as behavior is a common practice in many forms of CBT (e.g., dialectical behavior therapy), formulating cognition as feelings is less common. For instance, when clients “wrongly” affirm beliefs as feelings, the therapist may choose to use affective methods of change for beliefs rather than correcting the client (e.g., I feel nobody loves me is a thought, not a feeling, and we have to say I think/believe nobody loves me). Methods from emotion-focused therapy that help clients express getting in touch and process the feeling of *nobody loving them* may be used. A major advantage of the interdependence principle of Ellis‘ cognitive model is that therapists may use cognitive restructuring techniques that target new mechanisms that may be in control of cognition, such as behavioral factors (reinforcement, S-R association) or affective factors (emotional processing, verbal expression, labeling, incubation, and so on).

(2) Another implication is for the formulation process. The mainstream cognitive view assumes a mechanistic causation for the relationship between cognition and emotion (e.g., B determines C). As Barrett (2009) suggested, from a modal framework, the causation is probabilistic: being in state B increases the probability of being in state C (Barrett, 2009). In REBT terms, being in a psychological state of irrational cognition B (it's awful) increases the probability of having a psychological state of dysfunctional negative emotion (anxiety). Being in a state of rational cognition (it is bad, but not awful) increases the probability of having a psychological state of functional negative emotion (concern, not anxiety) (Tiba and Manea, 2018a).

(3) A focus on the specific grounding of beliefs may result in the diversification of the psychological interventions aimed at changing irrational beliefs. REBT considers rigid beliefs (e.g., “I must have the approval of significant others”) as core determinants of emotional disturbances. Although it is recognized that clients may use a verbal form of needs for expressing these beliefs, the grounded cognition approach suggests that they are grounded in sensitized brain systems involved in needs (Tiba, 2020). Changing factors that result in increases in states of need (deprivation perception, mental elaboration of deprivation and satisfaction, incubation) may help to change rigid beliefs.

Building on these assumptions, I illustrate how old ideas may bring new ways of understanding dysfunctional cognition and may advance the psychological treatment of emotional disorders.

## Author contributions

AT: Writing—original draft, Writing—review & editing.
